# The Impact of COVID-19 Outbreak on Nosocomial Infection Rate: A Case of Iran

**DOI:** 10.1155/2021/6650920

**Published:** 2021-02-25

**Authors:** Maryam Jabarpour, Mahlagha Dehghan, Giti Afsharipour, Elham Hajipour Abaee, Parvin Mangolian Shahrbabaki, Mehdi Ahmadinejad, Mahboobeh Maazallahi

**Affiliations:** ^1^Clinical Research Unit, Shahid Bahonar Academic Center, Kerman University of Medical Sciences, Kerman, Iran; ^2^Nursing Research Center, Kerman University of Medical Sciences, Kerman, Iran; ^3^Fellow of Critical Care Medicine, Kerman University of Medical Sciences, Kerman, Iran; ^4^Department of Critical Care Nursing, Kerman University of Medical Sciences, Kerman, Iran

## Abstract

**Background:**

Coronavirus disease-19 (COVID-19) is a new type of coronavirus that has caused a global pandemic. The disease is highly contagious, and all people are susceptible to the disease. Therefore, extensive measures were taken to prevent the spread of the disease at the community and hospitals. This study aimed to investigate the impact of COVID-19 outbreak on nosocomial infection rate.

**Methods:**

This cross-sectional study was conducted in an educational hospital, southeast Iran. The nosocomial infection rates of critical/intensive care units (CCU/ICUs) and medical-surgical units were assessed during and before the COVID-19 outbreak.

**Results:**

There was a 19.75-point decrease in the total rate of nosocomial infection during the COVID-19 outbreak (*P* = 0.02). In addition, there was a 39.12-point decrease in the total rate of CCU/ICUs' nosocomial infection during the COVID-19 outbreak (*P* < 0.001). A 19.23-point decrease was also observed in the total rate of medical-surgical units' nosocomial infection during the COVID-19 outbreak (*P* = 0.13). All kinds of CCU/ICUs' nosocomial infections had between 31.22- and 100-point decreases during the COVID-19 outbreak. Among medical-surgical units, 33.33- and 30.70-point decreases were observed only in UTI and SSI, respectively, during the COVID-19 outbreak, while BSI had a 40-point increase during the COVID-19 outbreak.

**Conclusions:**

Proper implementation of infection control protocols during the COVID-19 pandemic seems to reduce nosocomial infections.

## 1. Introduction

On February 11, 2020, the World Health Organization (WHO) identified the outbreak of a new coronavirus as the cause of a public health emergency worldwide, naming the disease caused by the novel coronavirus 2019 (2019-nCoV) as coronavirus disease-2019 (COVID-19) [1]. Most people with the disease had flu-like symptoms, but a number of people also developed severe respiratory complications and multiple organ failure, followed by death [2]. The mortality rate of the disease was approximately 6.6 percent at the time of the study [3]. According to the WHO, 18,354,342 confirmed cases with 696,147 deaths have been reported worldwide by August 2020 [4].

COVID-19 is highly contagious and is primarily transmitted through respiratory droplets and close contact, and all individuals are susceptible to the disease [5]. To this end, extensive measures and efforts were recommended to reduce the transmission of infection, such as keeping a safe distance, covering the mouth and nose with a tissue when coughing and sneezing, washing hands frequently, and using personal protective equipment such as face masks. The WHO and the Centers for Disease Control and Prevention (CDC) strongly recommended avoiding close and direct contact with people with acute respiratory infections, refraining from traveling to high-risk areas, and avoiding unprotected contact with domestic and wild animals [6]. For nosocomial infections to be prevented in health centers, infection control standards were used such as provision and proper distribution of equipment, triage strategy, congestion reduction, reorganization of wards and environmental health, and training and implementation of standard precautions such as safe injections, hand washing, and personal protective equipment [7].

Nosocomial infection occurs 48 to 72 hours after admission to other health care centers; the infection does not exist at the time of admission and is not in the incubation period [2, 8]. According to the WHO, 1.7 million nosocomial infections occur annually and 1 in 29 people develops nosocomial infections [9]. Globally, the incidence of these infections varies from 3.5% to 12% in developed countries and from 7.5% to 19.1% in low-income and middle-income countries [10]. The prevalence of nosocomial infections in Iran has been reported to be 4.5% [11]. These infections are mostly prevalent in the intensive care units (ICUs) and in older adults with underlying diseases and suppressed immune system [12]. Therefore, they cause several problems in the treatment course of patients, including the high cost of laboratory procedures, the use of medication, the length of hospital stay, and antibiotic resistance [13, 14]. This incident kills 99,000 people annually and imposes $20,000,000 on society [9].

Diagnostic, therapeutic, and invasive measures such as urinary catheter, central venous catheter, and mechanical ventilation are the factors that increase the incidence of nosocomial infections [15]. More than 80% of nosocomial infections include urinary tract infections, surgical site infections, respiratory infections, and bloodstream infections [14].

However, the widespread and rapid outbreak of COVID-19 is a major challenge for healthcare centers, and prevention and control of nosocomial infections is an important issue related to this disease. The most common way of transmission is through respiratory droplets and close contact with infected patients [16]. Prior to COVID-19, the WHO conducted an epidemiological study in 55 hospitals in 14 countries and found that the total mean prevalence of nosocomial infections was 8.7% [17]. Studies in the United States reported 36% of urinary tract infections (UTIs), 20% of surgical site infections (SSIs), 11% of bloodstream infections (BSIs) and pneumonia (PNEU), and 2.1% of nosocomial pneumonia with a mortality rate of 13.1% [12, 18]. Birgani et al. in Iran showed that 10% of patients admitted to the ICU had signs and symptoms of nosocomial infections, and in terms of infection type, 41% of UTI, 28% of respiratory infection, 20.5% of SSIs, and 10.5% of BSIs were reported [17]. Regarding the importance of nosocomial infections and pandemic of COVID-19 and the lack of a related study, this study was conducted to investigate the impact of COVID-19 outbreak on nosocomial infection rate.

## 2. Methods

### 2.1. Study Design and Setting

A cross-sectional design was used to investigate the nosocomial infection rate during and before COVID-19 outbreak in an educational hospital in southeast Iran. Bahonar hospital is the first and largest trauma center in the southeast of Iran. This hospital has 350 beds with the most advanced equipment and multiple wards. Its services include picture archiving and communication systems, telemedicine, flow cytometry, and the most advanced multiple neuromonitoring devices, and various surgeries such as cerebrovascular and brain aneurysm are done.

### 2.2. Sample Size and Sampling

The study sample was all data related to the nosocomial infection rate of critical/intensive care units and medical-surgical units in the first four months of Iranian new year (i.e., 2020-3-20 to 2020-7-21) which was during the COVID-19 outbreak and before the COVID-19 outbreak (i.e., 2019-3-20 to 2019-7-21). The inclusion criteria were all patients' files related to different nosocomial infections including ventilator-associated events (VAEs), pneumonia, urinary tract infection, bloodstream infection, and surgical site infection, and others. Incomplete and inaccurate data were excluded from the study.

### 2.3. Measurement

Infection control was evaluated based on the Iranian nosocomial infection surveillance system, which was launched in 2006. Data collection on nosocomial infections was performed by an infection control team consisting of a nurse and an infection control physician. In this method, the results of paraclinical tests were screened after the patient's admission and changes in the clinical condition.

In this method, the infection control nurse, in addition to his/her visits and observations, received daily reports from the head nurses (observation of signs such as fever, surgical site changes resulted from infection, change and initiation of new antibiotics, and ward physician's viewpoint of nosocomial infections) and daily culture results from the laboratory supervisor. Then, a diagnostic form was completed for each patient suspected of infection (based on the definitions of the Iranian nosocomial infection surveillance system) according to the hospital infection diagnosis algorithm (for events related to ventilator-associated events, pneumonia, UTI, bloodstream infection, surgical site infection, and other nosocomial infections) from the time of suspected infection until discharge or death of the patient. Forms approved by the infection control physician were submitted to the infection control committee of the hospital.

The following items in the diagnostic form were confirmed: ventilator-associated events (VAEs) based on clinical signs and radiological and microbiological findings, PNEU based on signs and symptoms of infection, culture results, and radiographic and serological findings, UTI based on signs and symptoms of the urinary tract, urine culture results, and ultrasonography, CT scan, and MRI, BSI based on signs and symptoms of the disease, blood culture results, and serological and imaging findings, and SSI based on signs and symptoms of the surgical site, culture results, and radiological findings. Finally, the rate of nosocomial infection at the hospital level was analyzed and reported by the infection control committee using the data of the diagnostic form.

### 2.4. Data Collection and Data Analysis

The researchers referred to the center after obtaining the code of ethics from the ethics committee. After getting permission from the head of the hospital, data related to a nosocomial infection rate of the CCU/ICUs (one CCU and four ICUs) and medical-surgical units (i.e., two orthopedic units, two neurosurgery units, two oncology wards, one urology ward, one general surgery ward, one general medical ward, and one maxillofacial ward) were gathered from the hospital infection control unit.

SPSS 20 was used for data analysis. Frequency, percentage, and confidence interval were used to describe the nosocomial infection rates during and before the COVID-19 outbreak. Percentage change (((new value − old value)/old value) *∗* 100) was used to describe changes in the nosocomial infection rates during and before the COVID-19 outbreak in CCU/ICCUs, medical-surgical units, and the total sample. Pearson's chi-square test was used to compare the nosocomial infection rate before and during the COVID-19 outbreak. The significance level was set at <0.05.

### 2.5. Ethical Considerations

The Kerman University of Medical Sciences approved the study protocol (IR.KMU.REC.1398.581).

## 3. Results

The total hospital admissions were 6135 cases in the first four months of the new Iranian year during the COVID-19 outbreak (i.e., 2020-3-20 to 2020-7-21), which were 17.72% less than those at the same time in the past year (i.e., 2019-3-20 to 2019-7-21). The total rate of nosocomial infection was 3.7% in the first four months of the new Iranian year during the COVID-19 outbreak, which was 19.57% less than the same time in the past year (*P* = 0.02). Except for VAE, pneumonia, and other infections, the rates of UTI and SSI decreased significantly compared with the same time in the past year (*P* < 0.05). A 12.5-point increase was observed in the rate of BSI during the COVID-19 outbreak compared with the past year; however, this was not statistically significant (Table 1).

The total critical/intensive care units' admissions were 858 cases in the first four months of the new Iranian year during the COVID-19 outbreak (i.e., 2020-3-20 to 2020-7-21), which were 12.7% more than those at the same time in the past year (i.e., 2019-3-20 to 2019-7-21). The total rate of CCU/ICUs nosocomial infection was 13.8% in the first four months of the new Iranian year during the COVID-19 outbreak, which had a 39.21-point decrease compared with the same time in the past year (*P* < 0.001). All kinds of CCU/ICUs nosocomial infections had between 31.22- and 100-point decreases during the COVID-19 outbreak compared with the same time in the past year. However, the changes were statistically significant about VET and other infections (*P* < 0.05) (Table 2).

The total medical-surgical care units' admissions were 5277 cases in the first four months of the new Iranian year during the COVID-19 outbreak (i.e., 2020-3-20 to 2020-7-21), which were 21.32% less than those at the same time in the past year (i.e., 2019-3-20 to 2019-7-21). The total rate of nosocomial infection in medical-surgical units was 2.1% in the first four months of the new Iranian year during the COVID-19 outbreak, which had a 19.23-point decrease compared with the same time in the past year; however, the changes were not significantly different (*P* = 0.13). Although UTI and SSI had 33.33- and 30.77-point decreases, respectively, during the COVID-19 outbreak compared with the same time in the past year, the changes were not statistically significant (*P* > 0.05). There were no percentage changes in VAE and other infections during the COVID-19 outbreak compared with the same time in the past year. BSI had a 40-point increase during the COVID-19 outbreak compared with the same time in the past year (Table 2).

## 4. Discussion

The results of the present study showed a statistically significant reduction in the rate of nosocomial infections during the COVID-19 outbreak. Adherence to infection control protocols is one of the most important factors in preventing nosocomial infections [19]. A study in New York found a reduced incidence of nosocomial infections by applying contact precautions [20]. Proper training and observance of infection control protocols during an epidemic of infectious diseases such as COVID-19 will reduce the rate of nosocomial infections. Infection control standards in the COVID-19 outbreak include contact, droplet, and, in some cases, aerial precautions, isolation of infected patients, identification of infected patients and personnel, and finally elimination of infection using antimicrobial agents. In addition, raising awareness is one of the most effective ways in fighting nosocomial infections, and the number of infections will decrease by continuing education and raising awareness and using effective and appropriate methods of infection control [15].

While the number of patients admitted to CCU/ICUs is less than that of other wards, the incidence of nosocomial infections is higher due to the long duration of admission and the implementation of various procedures [13]. In the present study, despite the increase of admission to CCU/ICUs during the corona epidemic, compared with the same time last year, there were between 31.25- and 100-point decreases in all types of nosocomial infections in CCU/ICUs. The results of a study in Egypt showed that educating staff on how to adhere to infection control standards reduced all nosocomial infections [12]. In addition, in a study on the ICU neuroprotection after an infection control program that included hand hygiene, contact precautions, isolation of infected patients, and disinfection of the environment, all nosocomial infections and antibiotic resistance reduced and patient's survival increased. However, at the beginning of the program, the rate of respiratory infections and UTIs increased, which was due to the identification and reporting of all cases of nosocomial infections following the initiation of the program [21].

The results of the present study showed a decrease in the total rate of infections compared with the previous year in the medical-surgical units. In addition, the rate of UTIs decreased, which is consistent with the study conducted by Meddings et al. According to Meddings et al.'s study, the use of infection control strategies significantly reduced the rate of UTIs [22]. Although the rate of SSI is higher in developing countries due to operating room congestion, poor hand hygiene, unnecessary use of antibiotics, etc. [23], in the present study, a decrease in the rate of SSI was observed, which is consistent with a study conducted by Jayasree and Afzal in 2019. According to Jayasree and Afzal's study, adherence to the infection control guideline reduced the rate of SSI [24].

In the present study, the rate of PNEU and VAEs in the medical-surgical units did not change, so there was no evidence of PNEU in the medical-surgical units before and during COVID-19. The purpose of infection control is to prevent cross transmission of pathogens, which plays an important role in the development of nosocomial infections, including PNEU and VAEs. In addition, effective infection control strategies include medical team training, hand hygiene, and the use of personal protective equipment [25]. The results of a review article in 2014 showed that the use of noninvasive mechanical ventilation, subglottic secretion drainage, and ventilator-related protocols such as oral hygiene and elevation of the head of the bed played important roles in the control of VAEs [26]. In addition, Salimi et al. showed that the rate of VAEs decreased because of the implementation of nursing care standards for standard oral care, proper handwashing, and proper staff training [27].

In the present study, the rate of BSI increased in medical-surgical units. The rate of BSI does not account for a large percentage, which can be due to the emphasis and importance of interventions related to blood catheters, which are considered in most medical centers [13]. Adherence to aseptic technique during catheter insertion, catheter care, and early catheter removal are examples of principles for preventing BSI [28]. Various studies showed a reduction in the total nosocomial infection as well as the rate of BSI with the implementation of hand hygiene, proper skin preparation, and catheter care [29, 30]. Furthermore, researchers found that the implementation of a problem-oriented training program, caregivers' awareness of the correct principles of infection control, and sterile conditions when implanting central venous catheters could play an important role in reducing BSI [31]. The increase in BSI in the present study may be due to the increase in antibiotic resistance and the increase in the duration of central venous catheter use.

The results of the present study showed a decreased number of admissions in the medical-surgical wards. Strict implementation of public health measures, which are undoubtedly crucial for controlling COVID-19, inadvertently affects health care systems. Furthermore, accurate instructions for staying at home and the fear of being infected in medical centers can also prevent people from going to the hospitals [32, 33].

The present study has some limitations. The study has been conducted in a trauma hospital, which is not a source hospital for patients with COVID-19. Therefore, the rates of nosocomial infections in hospitals admitting patients with COVID-19 may be different. Therefore, the generalizability of the results should be done with caution and it should be considered in future studies. Another limitation was the time of study at the early months of COVID-19, when health protocols were observed better and the risk of nosocomial infections reduced.

## 5. Conclusions

Proper implementation of infection control protocols during the COVID-19 outbreak reduces the rate of nosocomial infection. Therefore, nosocomial infections can be minimized by increasing awareness, creating a positive attitude, improving staff hygiene behaviors, and providing facilities to comply with the standards of infection control protocols.

## Figures and Tables

**Table 1:** Nosocomial infection rate before and during COVID-19 outbreak.

Time						
Variable	The first four months of the new year (before COVID-19) (*n* = 7454)		The first four months of the new year (during COVID-19) (*n* = 6135)		Percentage changes	Chi-square test (*P* value)
	*N* (%)	95% confidence interval	*N* (%)	95% confidence interval		
Ventilator-associated events (VAEs)	106 (1.4)	1.2–1.7	83 (1.4)	1.1–1.6	—	0.18 (0.73)
Pneumonia (PNEU)	2 (0.0)	0.0–0.1	0	—	—	—
Urinary tract infection (UTI)	59 (0.8)	0.6–1.0	31 (0.5)	0.3–0.7	−37.5	4.19 (0.04)
Bloodstream infection (BSI)	57 (0.8)	0.6–1.0	54 (0.9)	0.7–1.1	12.5	0.55 (0.46)
Surgical site infection (SSI)	102 (1.4)	1.1–1.6	57 (0.9)	0.7–1.2	−35.71	5.62 (0.02)
Others^*∗*^	15 (0.2)	0.1–0.3	5 (0.1)	0–0.2	−50	3.28 (0.07)
Total	341 (4.6)	4.1–5.1	230 (3.7)	3.3–4.3	−19.57	5.70 (0.02)

^*∗*^Meningitis or abscess.

**Table 2:** The nosocomial infection rate before and during COVID-19 outbreak in critical care units and medical-surgical units.

Time							
Variable		The first four months of the new year (before COVID-19) (*n* = 7454)		The first four months of the new year (during COVID-19) (*n* = 6135)		Percentage changes	Chi-square test (*P* value)
		*N* (%)	95% confidence interval	*N* (%)	95% confidence interval		
Ventilator-associated events (VAEs)	Critical care units	103 (13.8)	11.2–16.3	81 (9.4)	7.6–11.4	−31.88	7.22 (0.007)
	Medical-surgical units	3 (0.04)	0.0–0.1	2 (0.04)	0.0–0.1	—	0.03 (0.86)

Pneumonia (PNEU)	Critical care units	2 (0.3)	0.0–0.7	0	—	−100	—

Urinary tract infection (UTI)	Critical care units	17 (2.3)	1.2–3.3	9 (1.0)	0.5–1.7	−56.52	3.72 (0.054)
	Medical-surgical units	42 (0.6)	0.4–0.8	22 (0.4)	0.3–0.6	−33.33	2.44 (0.12)

Bloodstream infection (BSI)	Critical care units	24 (3.2)	1.9–4.7	19 (2.2)	1.3–3.3	−31.25	1.48 (0.22)
	Medical-surgical units	33 (0.5)	0.3–0.7	35 (0.7)	0.5–0.9	40	1.53 (0.22)

Surgical site infection (SSI)	Critical care units	14 (1.9)	0.9–2.9	7 (0.8)	0.2–1.5	−57.89	3.42 (0.06)
	Medical-surgical units	88 (1.3)	1.0–1.6	50 (0.9)	0.7–1.2	−30.77	3.46 (0.06)

Others^*∗*^	Critical care units	10 (1.3)	0.5–2.3	2 (0.2)	0.0–0.6	−84.62	6.53 (0.01)
	Medical-surgical units	5 (0.1)	0.0–0.1	3 (0.1)	0.0–0.1	-	0.14 (0.71)

Total	Critical care units	170 (22.7)	19.6–25.8	118 (13.8)	11.5–16.0	−39.21	21.51 (<0.001)
	Medical-surgical units	171 (2.6)	2.2–2.9	113 (2.1)	1.8–2.5	−19.23	2.35 (0.13)

^*∗*^Meningitis or abscess.

## Data Availability

Data are available from the corresponding author upon request by e-mail.
